# Acute-phase plasma proteomics of rabbit lung VX2 tumors treated by image-guided microwave ablation

**DOI:** 10.3389/fonc.2024.1435256

**Published:** 2024-08-26

**Authors:** Lin Cheng, Jin-zhao Peng, Sheng-wei Li, Zhi-xin Bie, Xiao-Guang Li

**Affiliations:** ^1^ Department of Minimally Invasive Tumor Therapies Center, Beijing Hospital, National Center of Gerontology, Institute of Geriatric Medicine, Chinese Academy of Medical Sciences, Beijing, China; ^2^ Medical School, University of Chinese Academy of Sciences, Beijing, China

**Keywords:** lung cancer, DIA, proteomics, microwave ablation, rabbit lung VX2 tumors

## Abstract

**Purpose:**

To explore the plasma proteomic changes of rabbit lung VX2 tumors treated by microwave ablation, and to explore the molecular pathway mechanisms that may be involved.

**Methods:**

New Zealand white rabbits were inoculated with VX2 tumor cell suspension in the right lower lung and treated with microwave ablation after 2-3 weeks of tumor formation. Blood was collected at 5 time points (TP1~TP5) before and after ablation by cardiac blood sampling and pre-treated before proteomic analysis. The plasma proteome was analyzed by Data-Independent Acquisition (DIA).

**Results:**

Different molecular pathways were activated at different time points:(i) TP1vsTP2: more proteins were down-regulated and enrichment analysis showed that the proteasome pathway was activated. The abnormal protein folding process involved in this pathway is closely related to the process of tumor development. (ii) TP2vsTP3: more proteins were up-regulated although the number of differentially differentiated proteins was lower and enrichment analysis showed that the phagosome pathway was activated. After microwave ablation inactivates tumor cells, it activates the phagosomal pathway for immune clearance of necrotic tumor tissue. (iii) TP3vsTP4: more down-regulated proteins, enrichment analysis showed that cysteine and methionine metabolism pathway was activated. Decreased metabolism of these amino acids suggests that cancer progression may be blocked after microwave ablation therapy. (iv) TP4vsTP5: the number of differential proteins was less and more down-regulated proteins, enrichment analysis showed that glutathione metabolism and metabolism of xenobiotics by cytochrome P450 pathway were activated. The down-regulated proteins in this pathway may suggest that microwave ablation may have reduced resistance to certain chemotherapeutic agents following.

**Conclusions:**

In the process of lung cancer treatment by microwave ablation, the changes of proteins on the possible molecular pathways at each time point are related to lung cancer, and not only involve some simple inflammatory reactions, and some of the proteins released by destroying the tumor cells can be used as possible drug binding sites and reduce drug resistance.

## 1 Introduction

Currently, lung cancer is still the leading cause of cancer deaths worldwide, and the incidence is increasing every year. According to the National Cancer Institute of the United States 2022, lung cancer is the leading cause of cancer death with a 5-year relative survival rate of 22.9% (1). Among them, non-small cell lung cancer (NSCLC) accounts for about 85% of lung cancer cases, and surgical treatment represented by lobectomy with hilar mediastinal lymph node dissection remains the gold standard for the treatment of early-stage NSCLC (2). For patients who do not tolerate surgical procedures, minimally invasive tumor therapy, as a less invasive treatment modality, has become an alternative therapy for this group of patients, including thermal ablation, cryoablation, and particle implantation. In the 2016 National Comprehensive Cancer Network NSCLC guidelines, thermal ablation was recommended as an alternative option for patients with non-surgical stage I NSCLC. Over the past decade, microwave ablation (MWA) has emerged as an advanced thermal ablation modality, offering higher temperatures, larger ablation zones, and shorter ablation times than radiofrequency ablation (3). While MWA continues to be used in current tumor therapy, acute phase changes in plasma proteomics are unknown.

The proteome can provide a more realistic and dynamic view of the molecular state of a patient’s state at a specific moment in time than DNA and RNA, and thus proteomics can be an effective tool for identifying diagnostic and therapeutic biomarkers for cancer (4, 5). Data-independent acquisition (DIA) is a targeted proteomics technique that can measure low abundance peptides, which can avoid the loss of large amounts of protein information and have higher quantitative accuracy and reproducibility. A lot of research has been done to study the relevant markers of lung cancer based on DIA proteomics technology, demonstrating different proteomics landscapes (6, 7). However, there is no report on what kind of proteomic landscape is demonstrated before and after thermal ablation and what cellular functions are involved, etc. In our study, we established a rabbit lung VX2 tumor model and performed microwave ablation, and collected rabbit plasma before modeling, after modeling, immediately after ablation, 4 days after ablation, and 7 days after ablation for proteomic analysis to compare the differences in protein profiles at different time points, and to dynamically present the changes in protein profiles, so as to provide possible reference targets for the efficacy, safety, and prognosis of ablation therapy for lung cancer.

## 2 Materials and methods

### 2.1 Materials

VX2 tumor cells are a transplantable rabbit squamous cell carcinoma line, which was provided by the Cell Resource Center, School of Basic Sciences, Peking Union Medical College, Institute of Basic Medical Sciences, Chinese Academy of Medical Sciences; New Zealand white rabbits were purchased from Beijing Keyu Animal Breeding Center (license number: SCXK (Beijing, China). 2018–0010); microwave ablator: ECO-100A1 MW ablation system (ECO Medical Instrument, Nanjing, China) was used, with a microwave emission frequency of 2450 ± 50 MHz and an adjustable continuous wave output power of 20–80 W. Disposable microwave ablation antenna: Nanjing Yigao, specification φ1.6×100mm, model ECO-100AI3, lot number 221017003; CT machine: CT590; GE Healthcare, Pittsburgh, Pennsylvania.

### 2.2 Establishment of a rabbit lung VX2 tumor model

All operations were performed under aseptic conditions. After the frozen VX2 cell suspension was resuscitated by conventional cell culture method, 1 ml (10^7^·ml^-1^) was taken and inoculated on the medial muscle tissue of the hind leg of tumor-bearing rabbits, and the tumor was formed after 2–3 weeks, and the fish-like tissues with vigorous growth on the edge of the tumor mass were taken, sheared into a puree, digested with trypsin and passed through a mesh sieve, and made into a cell suspension by using PBS solution, and 1 ml (10^7^·ml^-1^) cell suspension was extracted with a 1ml syringe.

New Zealand female rabbits (weight 2.5–3.0 kg) were injected with 10% chloral hydrate (1.5 ml/Kg) via a marginal ear vein for general anesthesia, and a 21G puncture needle was punctured into the lower lobe of the right lung of the rabbits after connecting to a 1 ml syringe for injection of the VX2 tumor cell suspension under CT guidance (1.25 mm layer thickness, 120 kV tube voltage, 200 mA tube current). Microwave ablation was performed after 2–3 weeks when the tumor diameter was about 10 mm by CT scan.

In this experiment, 10 out of 12 New Zealand white rabbits were successfully modeled, with a modeling success rate of 83.33%. When isolated lesions appeared in the lungs of the experimental rabbits, and the model was successfully built. The tumor layer with the largest cross-sectional area in the CT group image of each rabbit was selected, and the tumor diameter was measured. The tumor lesions were located in the right lower lung of the rabbits. After successfully establishing VX2 lung tumor models in 10 rabbits, we set up five time points for observation. Two rabbits died before the final time point, leaving us with 10 rabbits for the first three time points and 8 rabbits for the last two time points. The mean tumor diameter was 11.45 ± 1.65 mm (range 9.33–13.69 mm, median 12.72 mm).

### 2.3 Microwave ablation

Under CT guidance, when the ablation antennas was successfully punctured to the lesion, we connected the microwave ablation antennas to the microwave ablation device, confirmed that the circulating water cooling system was opened, set the ablation power of 20 w and the ablation time of 2 min, and started the microwave ablation device. During the ablation period, the state of the experimental rabbits was observed at all times. After ablation, the ablation antenna was withdrawn and a CT scan was performed again to observe whether there were any complications such as pneumothorax, hemorrhage and pleural effusion. If pneumothorax was present and lung compression was more than 20%, then perform thoracic aspiration.

**Figure 1:**
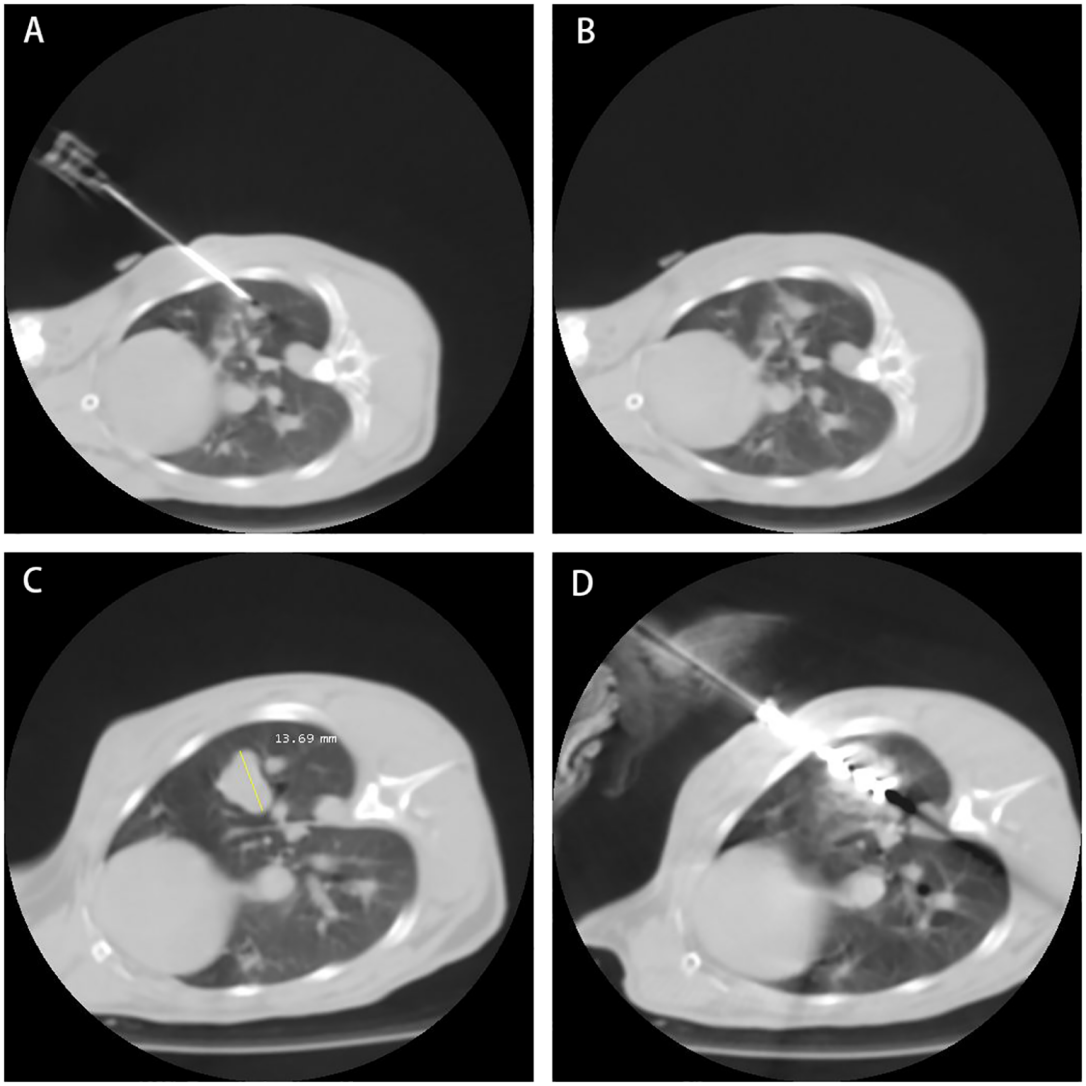
The process of rabbit lung ablation. **(A)** Establishing the Model: Establishing the rabbit VX2 tumor model: Implant VX2 tumor cells into the rabbit. **(B)** Model Established: Model established: Successful implantation of VX2 tumor cells confirmed. **(C)** Model at Third Week: Third week post-implantation: Monitoring tumor growth and development. **(D)** Microwave Ablation of Rabbit Lung VX2 Tumor: Microwave ablation of rabbit lung VX2 tumor: Treatment procedure to ablate the tumor.

All rabbit lung VX2 tumor ablation treatments were performed by physicians with more than 5 years of experience in tumor ablation. The process is shown in **Figure 1**.

### 2.4 Rabbit plasma specimens

In this study, the sampling method was cardiac blood sampling, and blood samples were collected at the following five time points (TP)-before establishing the model (TP1), model established (TP2), immediately after ablation (TP3), 4 days after ablation (TP4), and 7 days after ablation (TP5). All samples were processed according to standardized operating procedures. Blood collected in EDTA anticoagulated tubes was centrifuged at room temperature (3000 rpm, 10 min) within 2 hours after cardiac puncture, and the supernatant was transferred to clean centrifuge tubes, 0.2 mL per tube, and frozen in a -80°C refrigerator.

### 2.5 DIA-based proteomics analysis

Sample Preparation: Acquired plasma samples were pretreated to remove any contaminants and proteins were extracted. Protein concentration was determined using BCA assay. Extracted proteins were then subjected to reduction, alkylation, and enzymatic digestion. Proteins are reduced with 10mM dithiothreitol (DTT) to break disulfide bonds, alkylated with 5mM iodoacetamide (IAA) to prevent reformation of disulfide bonds. Each sample was subjected to filter-aided sample preparation (FASP) enzymatic digestion method using an appropriate amount of protein (8).

Mass Spectrometry Analysis: Peptides were analyzed using a Thermo Scientific Q Exactive HF mass spectrometer. The mass spectrometer was operated in Data-Independent Acquisition (DIA) mode. In DIA mode, the mass spectrometer sequentially scans predefined mass ranges (350–1150 m/z) to acquire comprehensive and unbiased peptide spectra (9).

Data Processing and Analysis: The acquired DIA data were processed using Spectronaut (Biognosys) software. The software was configured with specific search parameters: the Uniprot rabbit database. The peptide and protein false discovery rate (FDR) was kept below 1.0%, with each protein identified by at least one specific peptide. Quantification was performed at MS2 level using label-free quantification (LFQ), with default settings for other parameters (10).

## 3 Result

### 3.1 Quantitative proteomics analysis

**Figure 2:**
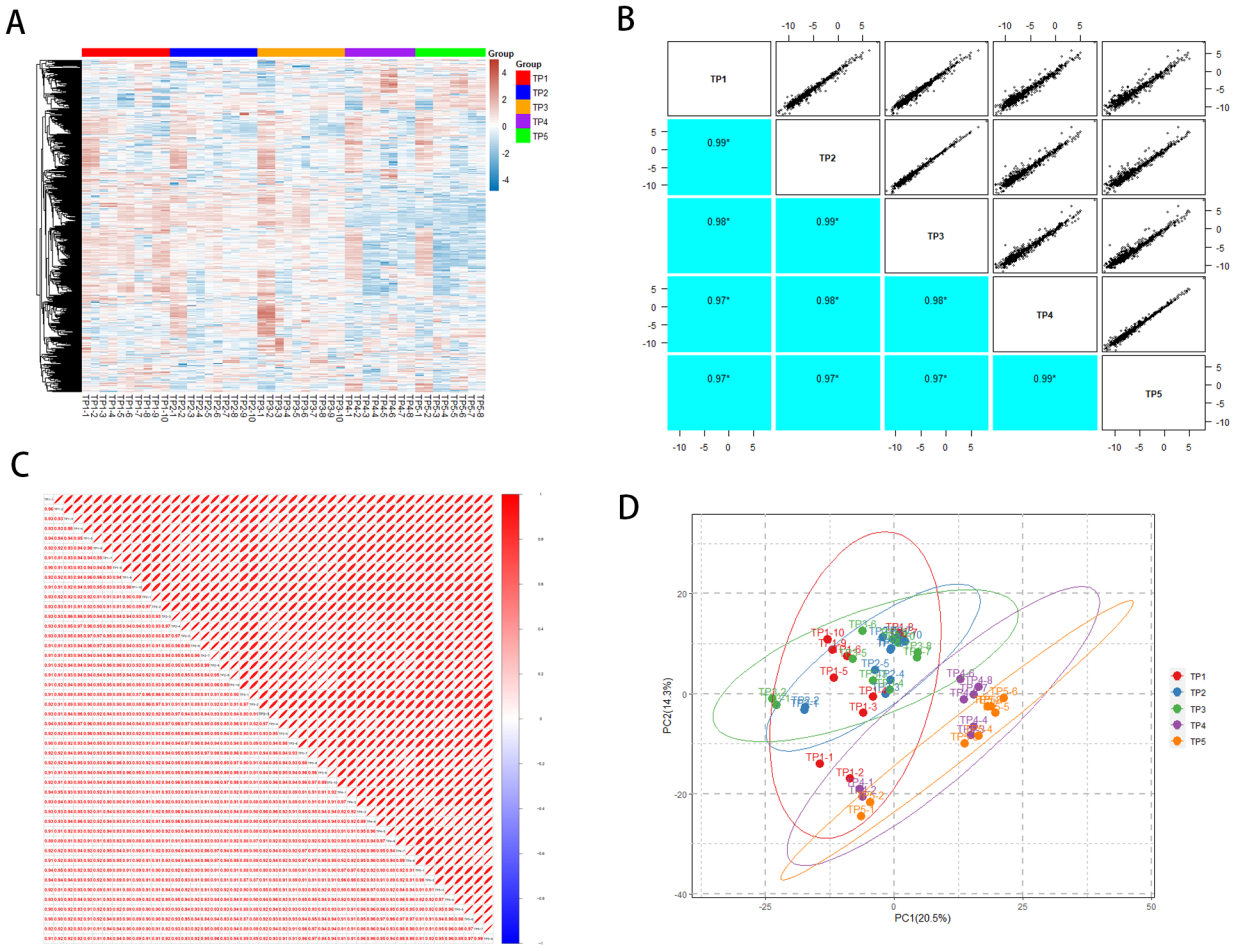
General overview of protein identification. **(A)** Heatmap of TP1~TP5 proteome distribution. **(B)** TP1~TP5 intergroup correlations. **(C)** Correlation between TP1~TP5 samples. **(D)** Principal component analysis of TP1~TP5.

Using DIA-based proteomics technology, we systematically monitored the plasma protein expression profiles of rabbit lung tumors in the acute phase after ablation therapy. The identification process in this study was to first degrade the proteins into peptides, use mass spectrometry for peptide identification, and later deduce the possible proteins; we detected a total of 16,982 peptides and identified 1,120 proteins (**Supplementary Material 1**). In order to assess the overall situation of the obtained proteomic data and to ensure the stability of the mass spectrometry analysis and the accuracy of the DIA quantification results, we evaluated the effect of mass spectrometry detection at both the peptide and protein levels. A total of 758 different proteins were quantified for each sample, and the distribution of the quantification values of all proteins for each sample was demonstrated by using a heat map (**Figure 2A**), and correlation analyses were carried out (**Figures 2B, C**), which showed that the correlation of the samples between sample groups TP1~TP5 was high (DIA sequencing data is eligible > 0.9), and that multiple samples within the same group had a better stability. In addition, we performed principal component analysis (PCA) on the samples at five time points (**Figure 2D**), which showed the general changes in plasma protein profiles before and after MWA.

### 3.2 Plasma proteomics differential protein analysis

Differential proteins were screened according to the multiplicity of difference in protein quantification values (1.5-fold and above) and statistical significance (P ≤ 0.05). The quantitative protein data of TP1 to TP5 were subjected to grouped t-tests, and the number of differential proteins and the number of up-regulated/down-regulated proteins compared between groups are shown in **Table 1**, and the specific differential proteins are shown in **Supplementary Material 2**.

**Table 1:** Number of differentially expressed proteins in groups.

	Differential proteins	Up- regulated proteins	Down- regulated proteins
**TP1 vs TP2**	179	53	126
**TP2 vs TP3**	48	46	2
**TP3 vs TP4**	183	46	137
**TP4 vs TP5**	53	7	46

**Figure 3:**
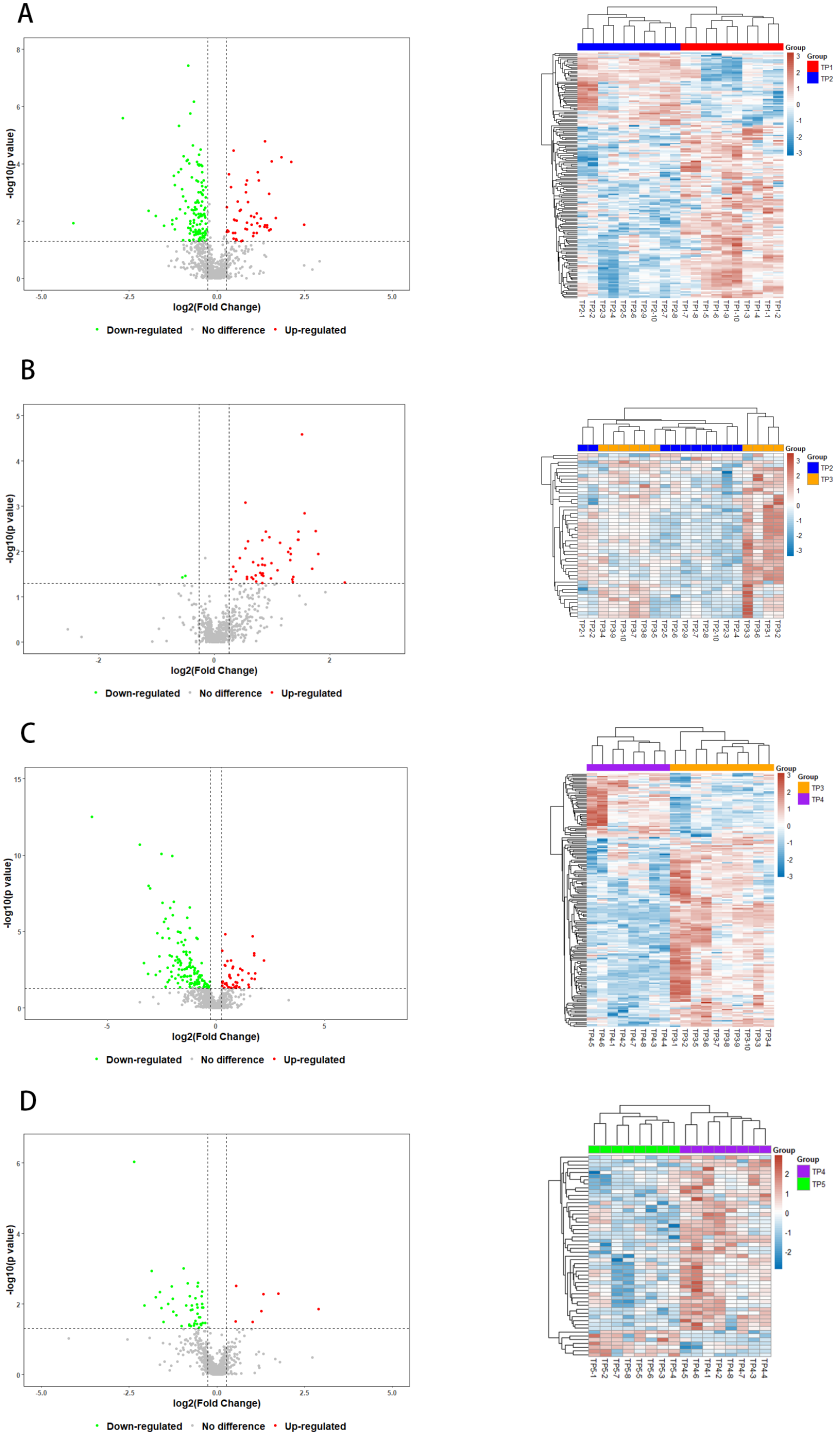
Volcano plots and Heatmaps of Differential Proteins. Red and green points indicated up-regulated and down-regulated proteins, respectively. **(A)** Volcanograms and Heatmaps of TP1vsTP2 Differential Proteins, **(B)** Volcano plots and Heatmaps of TP2vsTP3 Differential Proteins, **(C)** Volcano plots and Heatmaps of TP3vsTP4 Differential Proteins, **(D)** Volcano plots and Heatmaps of TP4vsTP5 Differential Proteins.

By using the protein expression ratio (i.e., fold difference) and the t-test significance index-p-value, a volcano plot can be plotted, where the horizontal coordinate is the logarithm of the ratio with a base of 2, and the vertical coordinate is the negative logarithmic transformation of the p-value with a base of 10, to visualize the screened differential proteins (**Figure 3**). The heatmap visualizes the distribution of quantitative and statistical values of all differential proteins and shows the distribution of differential proteins (**Figure 3**).

### 3.3 Functional annotation analysis of differential proteins

**Figure 4:**
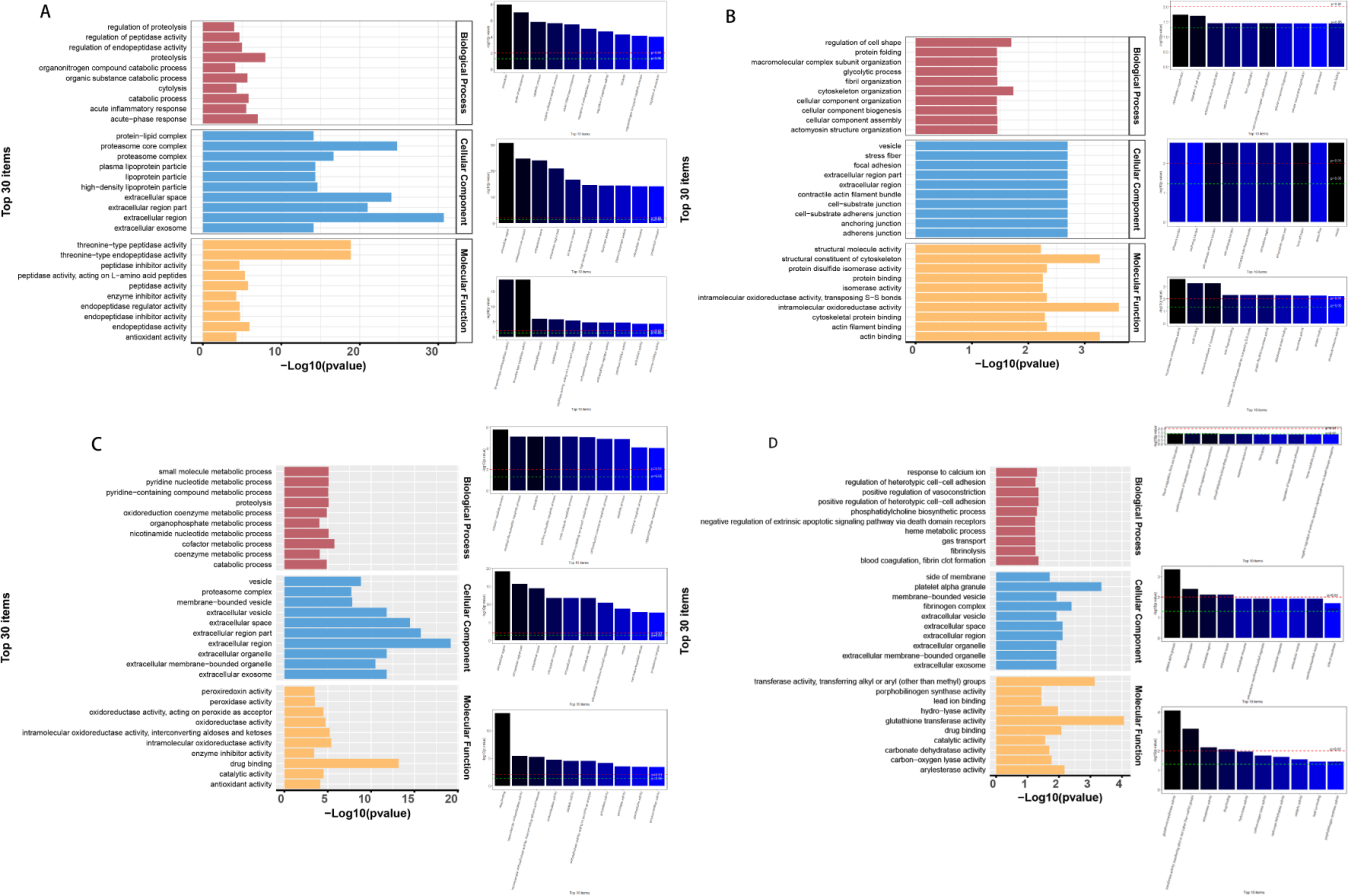
GO enrichment analysis of differential proteins. **(A)** GO analysis of TP1vsTP2 differential proteins, **(B)** GO analysis of TP2vsTP3 differential proteins, **(C)** GO analysis of TP3vsTP4 differential proteins, **(D)** GO analysis of TP4vsTP5 differential proteins.

The differential proteins screened for TP1vsTP2, TP2vsTP3, TP3vsTP4, and TP4vsTP5 were subjected to GO analysis (http://www.geneontology.org/) with p-values, and the top 30 GO functional entries enriched (sorted in ascending order by -LOG10P) were plotted. Differential proteins for TP1vsTP2 were mainly enriched in (i) CCs: extracellular region, proteasome core complex and extracellular space; (ii) MFs: threonineulare endopeptidase activity and threoninedasee peptidase activity; and (iii) BPs: proteolysis, acuteolysis response and catabolic process (**Figure 4A**). Differential proteins for TP2vsTP3 were mainly enriched in (i) CCs: adherens junction, anchoring junction and celltiongialee adherens junction; (ii) MFs: intramolecular oxidoreductase activity, actin binding and structural constituent of cytoskeleton; and (iii) BPs: cytoskeleton organization and regulation of cell shape (**Figure 4B**). Differential proteins for TP3vsTP4 were mainly enriched in (i) CCs: extracellular region, extracellular region part and extracellular space; (ii) MFs: drug binding, intramolecular oxidoreductase activity and intramolecular oxidoreductase activity, interconverting aldoses and ketoses; and (iii) BPs: cofactor metabolic process, nicotinamide nucleotide metabolic process and proteolysis (**Figure 4C**). Differential proteins for TP4vsTP5 were mainly enriched in (i) CCs: platelet alpha granule, fibrinogen complex and extracellular region; (ii) MFs: glutathione transferase activity, transferase activity, transferring alkyl or aryl (other than methyl) groups and arylesterase activity; and (iii) BPs: blood coagulation, fibrin clot formation and positive regulation of heterotypic cellrotyp adhesion (**Figure 4D**).

### 3.4 KEGG pathway analysis

**Figure 5:**
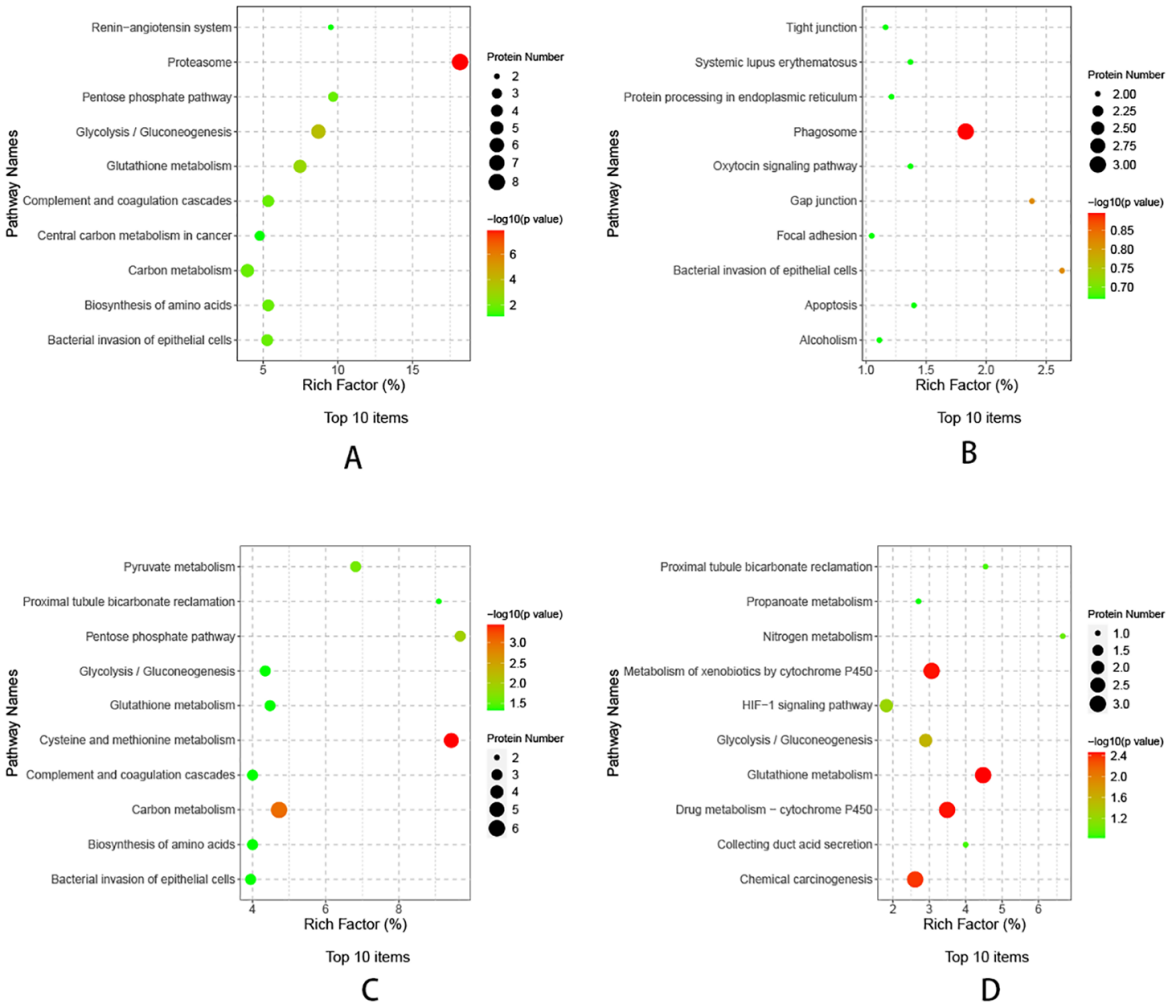
KEGG pathway analysis of differential proteins. **(A)** KEGG analysis of TP1vsTP2 differential proteins, **(B)** KEGG analysis of TP2vsTP3 differential proteins, **(C)** KEGG analysis of TP3vsTP4 differential proteins, **(D)** KEGG analysis of TP4vsTP5 differential proteins.

The differential proteins of TP1vsTP2, TP2vsTP3, TP3vsTP4, and TP4vsTP5 were enriched for the KEGG pathway, and bubble plots were made for the top 10 functional entries enriched (sorted in descending order according to the -LOG10PValue). The differential proteins of TP1vsTP2 were mainly enriched in proteasome pathway, the differential proteins of TP2vsTP3 were mainly enriched in phagosome pathway, the differential proteins of TP3vsTP4 were mainly enriched in cysteine and methionine metabolism pathway, the differential proteins of TP4vsTP5 were mainly enriched in glutathione metabolism and Metabolism of xenobiotics by cytochrome P450 pathway (**Figure 5**).

### 3.5 Reactome pathway enrichment analysis

Reactome (https://reactome.org/) is an open-source, open-access, manually curated, and peer-reviewed database for gene annotation data. The core unit of the Reactome data model is the reaction. The entities involved in reactions (nucleic acids, proteins, complexes, and small molecules) form networks of biological interactions and are grouped into pathways. For this analysis, a plot of the top 10 enriched functional entries will be created. The differential proteins of TP1vsTP2 were mainly enriched in Crosshedtialdome.o of soluble exogenous antigens (endosomes) pathway, the differential proteins of TP2vsTP3 were mainly enriched in Signaling by Rho GTPases, Miro GTPases and RHOBTB3 and Signaling by Rho GTPases pathway, the differential proteins of TP3vsTP4 were mainly enriched in Metabolism pathway, the differential proteins of TP4vsTP5 were mainly enriched in metabolism pathway.

### 3.6 Drawing of protein-protein interaction networks

**Figure 6:**
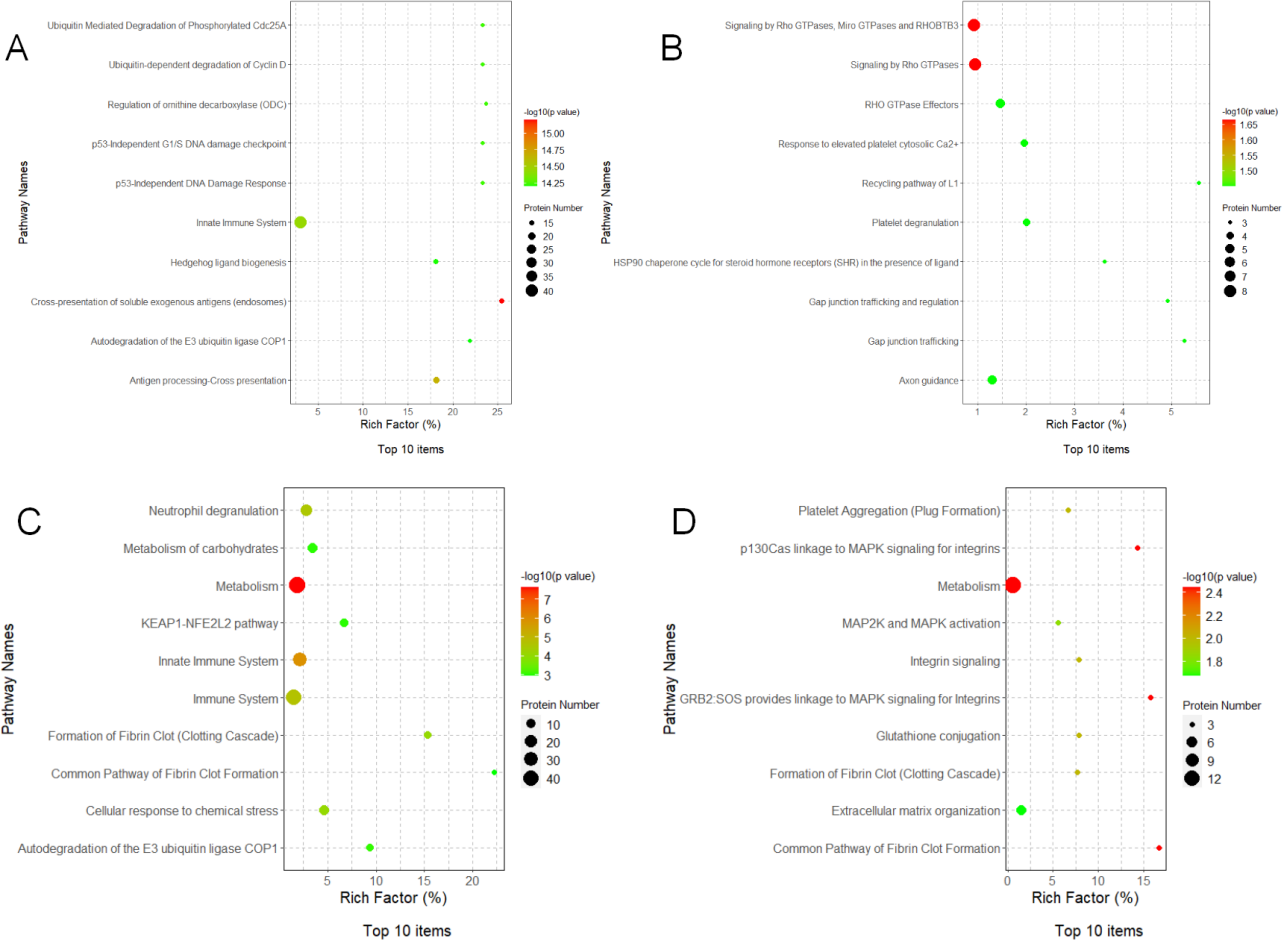
Reactome Pathway Enrichment Analysis of differential proteins. **(A)** Reactome Pathway of TP1vsTP2 differential proteins, **(B)** Reactome Pathway of TP2vsTP3 differential proteins, **(C)** Reactome Pathway of TP3vsTP4 differential proteins, **(D)** Reactome Pathway of TP4vsTP5 differential proteins.

**Figure 7:**
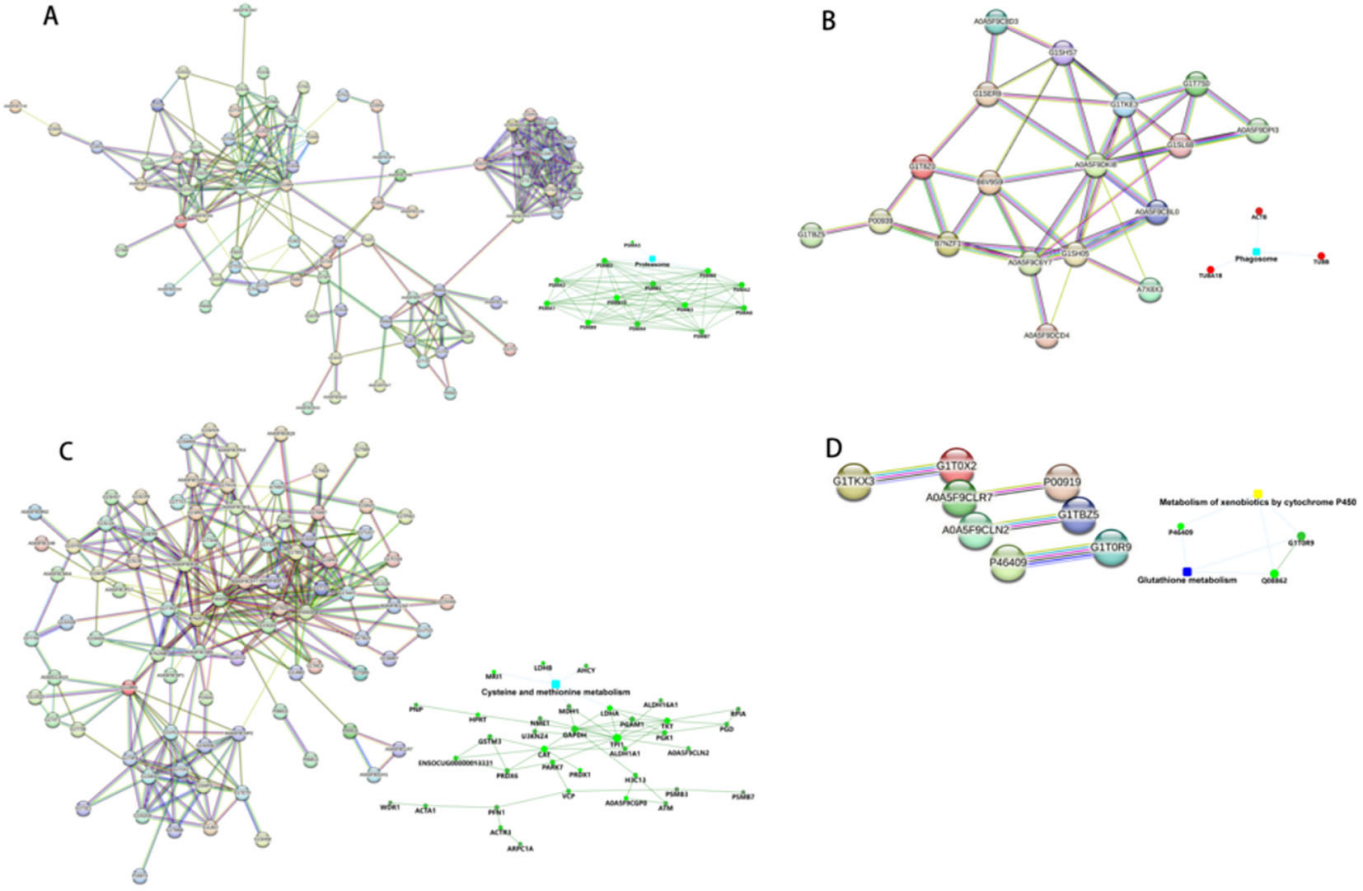
The differential proteins identified by PPI analysis. **(A)** PPI analysis of TP1vsTP2 differential proteins. **(B)** PPI analysis of TP2vsTP3 differential proteins. **(C)** PPI analysis of TP3vsTP4 differential proteins. **(D)** PPI analysis of TP4vsTP5 differential proteins.

The String 12.0 database (https://string-db.org/) is a database that searches for interactions between proteins. Protein interaction network maps were done for the differential proteins of TP1vsTP2, TP2vsTP3, TP3vsTP4, and TP4vsTP5, and the results are shown in **Figure 6**. We followed the results of KEGG pathway analysis to excerpt the different proteins on the proteasome pathway by TP1vsTP2, the phagosome pathway by TP2vsTP3, the cysteine and methionine metabolism pathway by TP3vsTP4, the Glutathione pathway and the Metabolism of xenobiotics by cytochrome P450 pathway by TP4vsTP5, as shown in **Figure 7**.

### 3.7 Differential proteins in multiple groups

**Figure 8:**
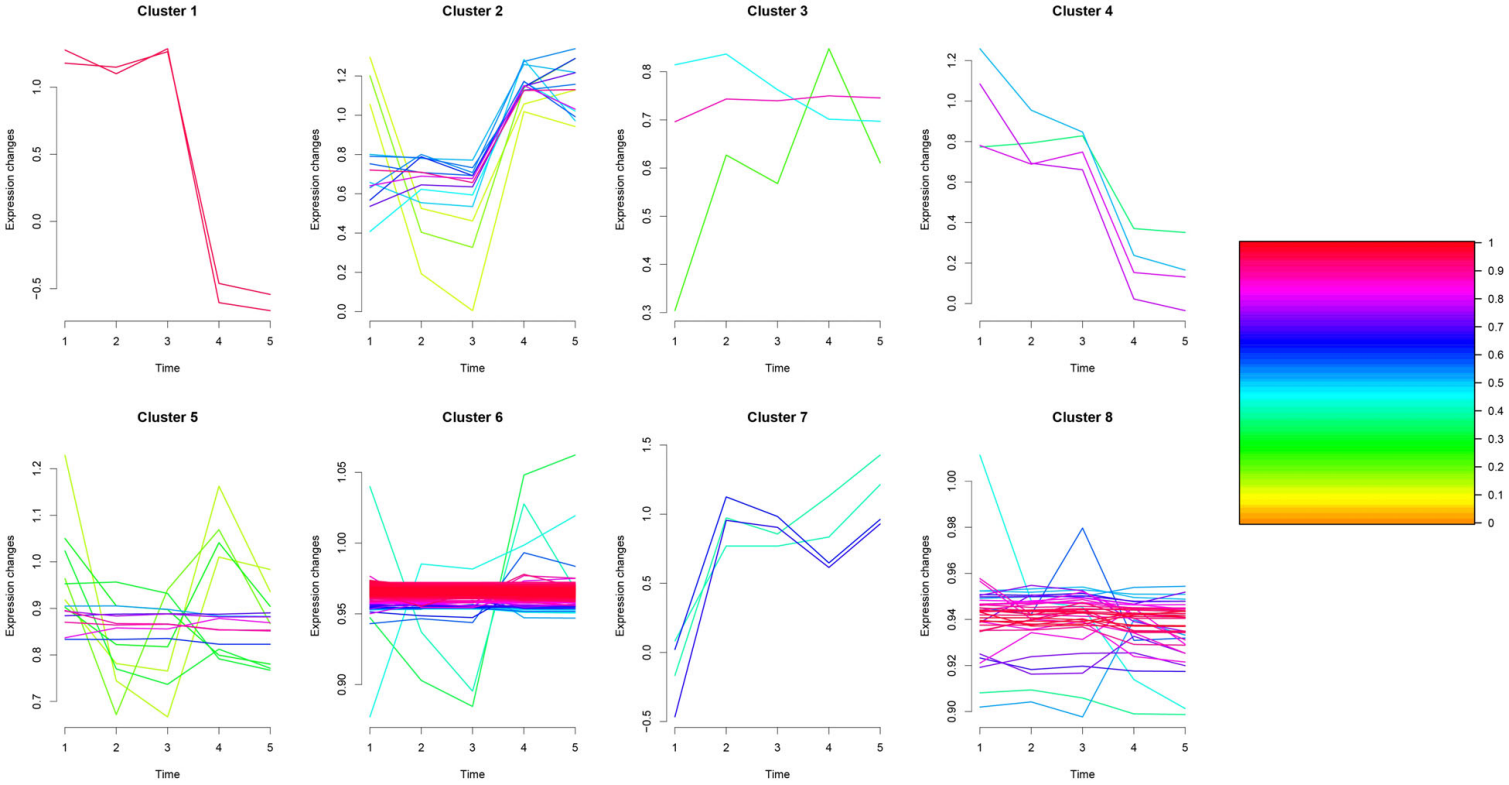
Multi-sample Mfuzz analysis plot.

One-way Analysis of Variance (one-way ANOVA) was performed on the samples of TP1~TP5 groups to screen for differential proteins with *p*-value ≤ 0.05, and a total of 268 differential proteins were identified. We utilized the Mfuzz package to cluster the differential protein expression profiles of TP1~TP5. Overall, we observed 8 different temporal pattern clusters representing differentially regulated proteins(**Supplementary Material 3** and **Figure 8**). We performed functional enrichment analysis for different clusters (**Supplementary Figures 13**–**20**).

## 4 Discussion

Thermal ablation, which aims to induce coagulative necrosis consistent with the tumor and its margins, has been used as the main treatment option for early-stage NSCLC in recent years (11, 12), especially for early-stage lung cancer with a tumor diameter of <3 cm, and it can effectively prolong the survival time of patients in the treatment of lung malignancies (13). In recent years, LC-MS-based proteomics has proven to be an extensive tool for plasma biomarker screening. All parent ions in the selected m/z range can be fragmented during DIA and analyzed in a single MS/MS scan. DIA has become a powerful approach in proteomics research, offering detailed, reproducible, and quantitative data for complex biological samples. Our study explored the acute-phase changes in the plasma proteome following microwave ablation of rabbit lung VX2 tumors.

The results showed that before and after inoculation of rabbit lungs with VX2 tumors-TP1vsTP2 were mainly involved in the biological processes of acute-phase response and catabolic process, indicating the response of the rabbit organism caused by the tumors in the course of growth. In this process, down-regulation of proteins mainly enriched in the proteasome pathway, such as PSMB10, PSMB3, PSMA1, PSMA2, etc. The proteasome is a highly organized and complex structure that is the core of the ubiquitin-proteasome system (UPS), including PSMBs and PSMAs.Dysregulation of the UPS has been closely associated with a variety of cancers (14, 15), and it has been suggested that PSMA1 may be a key gene regulating the development of LUSC (16). The UPS has now become a target for drug development (17), and a variety of inhibitors have been used to treat different forms of cancer (18, 19).

Before ablation and immediately after ablation -TP2vsTP3, the biological processes involved are cytoskeletal and cellular morphology changes, probably due to the ablation process leading to tumor tissue coagulation necrosis and tumor cell morphology changes. More proteins were upregulated in this process, such as ACTB, TUBB and TUBA1B, which mainly enriched in phagosomal pathway. All three proteins are housekeeping proteins. β-actin (ACTB), a highly conserved cytoskeletal structural protein associated with cell growth and cell migration (20), is up-regulated and aggregated which may promote tumor cell invasion (21). TUBB is essential for cell division and intracellular signaling and transduction (22). In lung cancer patients, TUBB expression is increased in tumor tissues compared to adjacent normal tissues, and higher expression of TUBB is associated with poorer overall survival (23). TUBA1B is associated with microtubule formation (24), and thus is involved in cancer cell progression and invasion. Serum TUBA1B protein levels were significantly higher in lung cancer patients than in healthy individuals, and higher expression of TUBA1B was significantly associated with shorter overall survival (25). After microwave ablation inactivates tumor cells, it releases housekeeping genes that activate phagosomal pathways for immune clearance of necrotic tumor tissue.

Immediately after ablation compared to 4 days after ablation-TP3vsTP4, the biological processes involved were mainly cofactor metabolism processes, it is worth mentioning that the molecular functions in this process were mainly related to drug binding, probably due to the release of tumor antigens from the necrotic tumor cells, which exposed some possible drug binding sites. Down-regulation of proteins was mainly observed, which mainly enriched in the cysteine and methionine metabolism pathway, with proteins such as LDHB, AHCY, MDH1, etc. Glucose metabolism is an important metabolic process for maintaining cellular energy. In the absence of oxygen, aerobic glycolysis converts glucose to lactate via lactate dehydrogenase (LDH), which has two common subunits, LDHA and LDHB. It has been shown that glycolytic metabolism enhances the antitumor activity of immune cells and may be used to improve cancer therapy (26). Malate dehydrogenase 1 (MDH1) is a critical enzyme in the catabolic pathway of glutamine catabolism, where methionine and cystine are the only amino acids involved in protein synthesis. As an essential amino acid, methionine must be ingested through the diet (27), whereas cystine is a semi-essential amino acid that can be obtained from the diet or synthesized *de novo* from methionine and serine through the transsulfuration pathway. One study found that a methionine/cystine restricted diet significantly blocked cancer progression (28). Microwave ablation inactivates the metabolic down-regulation of glucose and some amino acids that occurs in the acute phase after tumor inactivation, thus blocking the progression of lung cancer.

Compared with 4 days after ablation and 7 days after ablation - TP4 vs TP5, the main biological processes involved are blood coagulation, fibrin clot formation. More proteins were down-regulated, such as Q08862, GSTM3 and P46409 enriched in glutathione metabolism and metabolism of xenobiotics by cytochrome P450 pathway. It has been argued that GSTM3 has a protective role in small cell lung cancer (29), and that a decrease in its abundance predicts tumor recurrence and poor prognosis (30). Q08862 and P46409 are both fragments of Glutathione S-transferase (GST), an enzyme that binds glutathione as well as various endogenous and exogenous metabolites during biotransformation (31). Metabolism of xenobiotics by cytochrome P450 pathway may be related to the organism’s response to drugs (32).

There are shortcomings in rabbit plasma proteomics: (i) VX2 implant tumors are not the same as human lung cancer, and 2) rabbit proteomics are not necessarily representative of humans, because rabbit plasma is not as rich in proteins as that of human beings, and the results presented are limited.

## 5 Conclusions

Our study found that the protein changes in potential molecular pathways at each time point were associated with the development of lung cancer. These changes were not limited to simple inflammatory responses; rather, the complex mechanisms of these molecular pathways require further investigation. It is worth mentioning that our study found that the destruction of tumor cells after ablation resulted in the release of specific proteins associated with drug binding sites. These proteins may serve as potential targets for subsequent drug therapy.

## Data Availability

The datasets presented in this study can be found in online repositories. The names of the repository/repositories and accession number(s) can be found in the article/**Supplementary Material**.
