# Supplementary material for: Acute-phase plasma proteomics of rabbit lung VX2 tumors treated by image-guided microwave ablation
Source: Front Oncol. 2024 Aug 26;14:1435256. doi: 10.3389/fonc.2024.1435256 (PMC11381224; doi:10.3389/fonc.2024.1435256)
Supplement: Supplementary file 1 [file DataSheet1.doc]

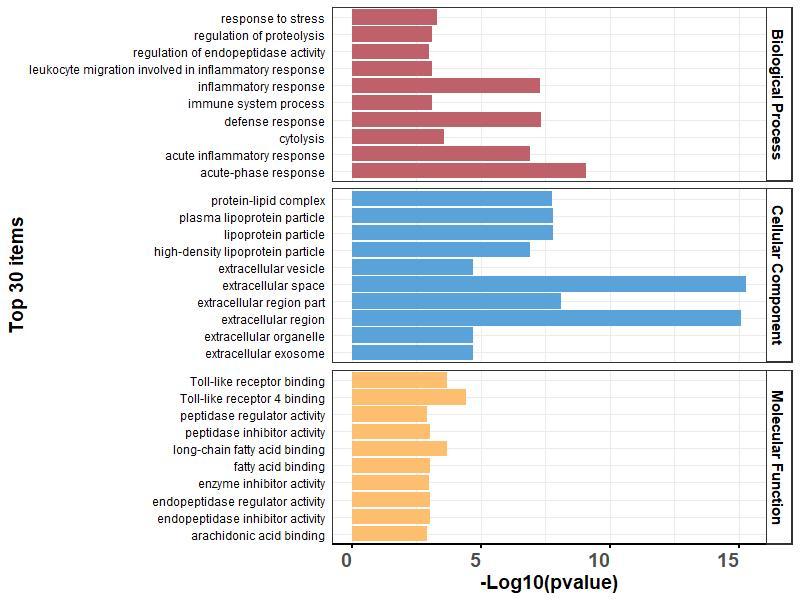

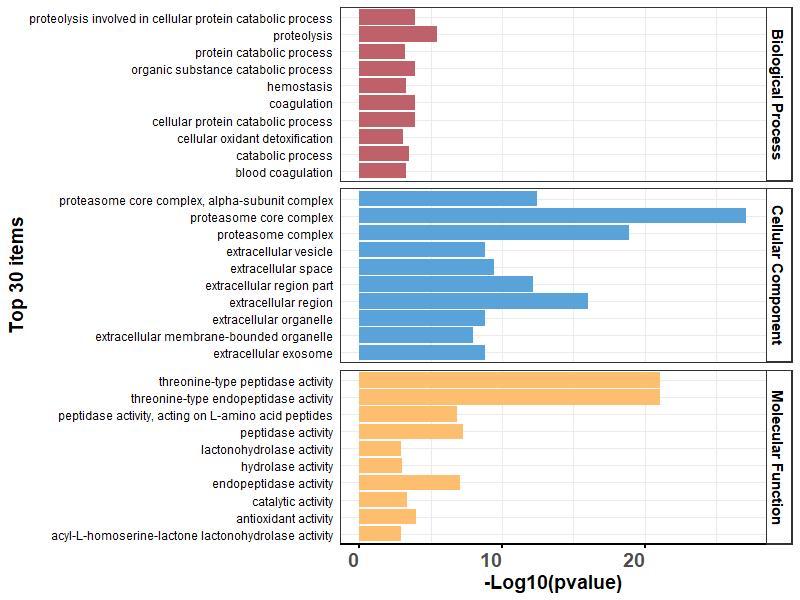


Figure 1 GO analysis of TP1vsTP2 up-regulated proteins, and GO analysis of TP1vsTP2 down-regulated proteins.


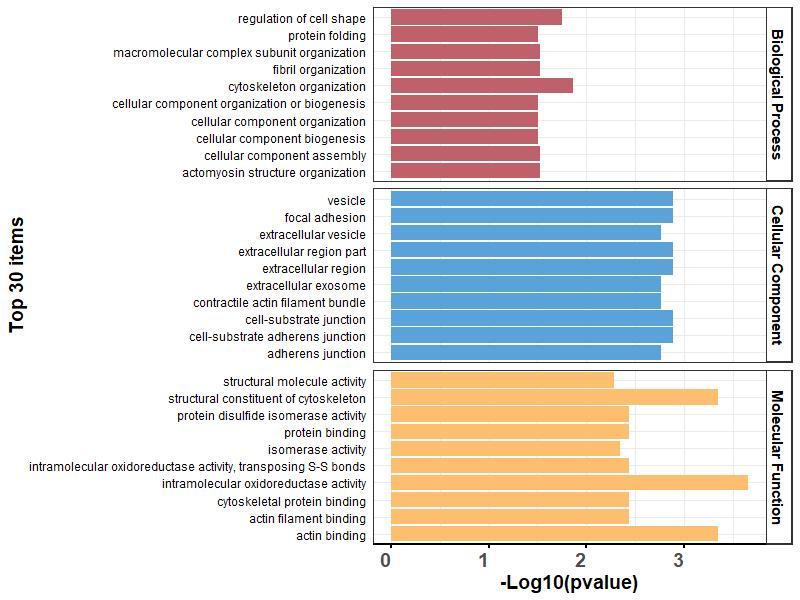

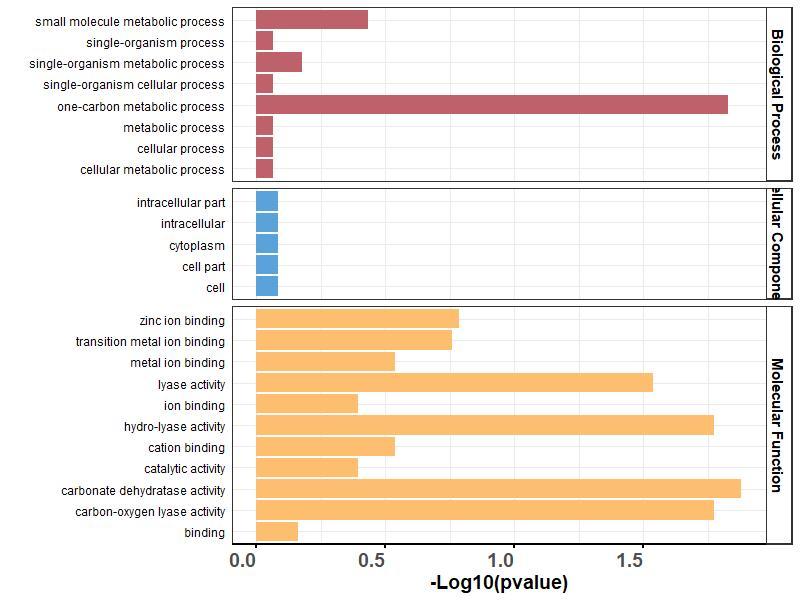


Figure 2 GO analysis of TP2vsTP3 up-regulated proteins, and GO analysis of TP2vsTP3 down-regulated proteins.


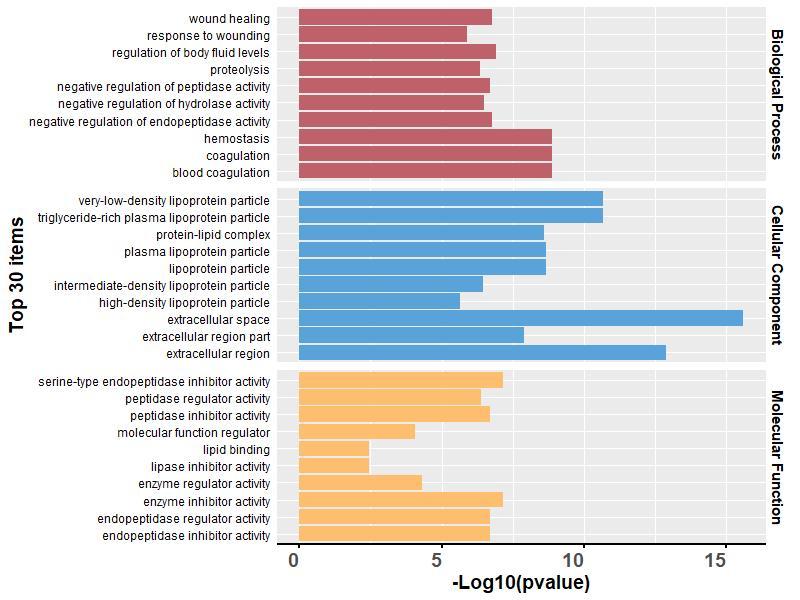

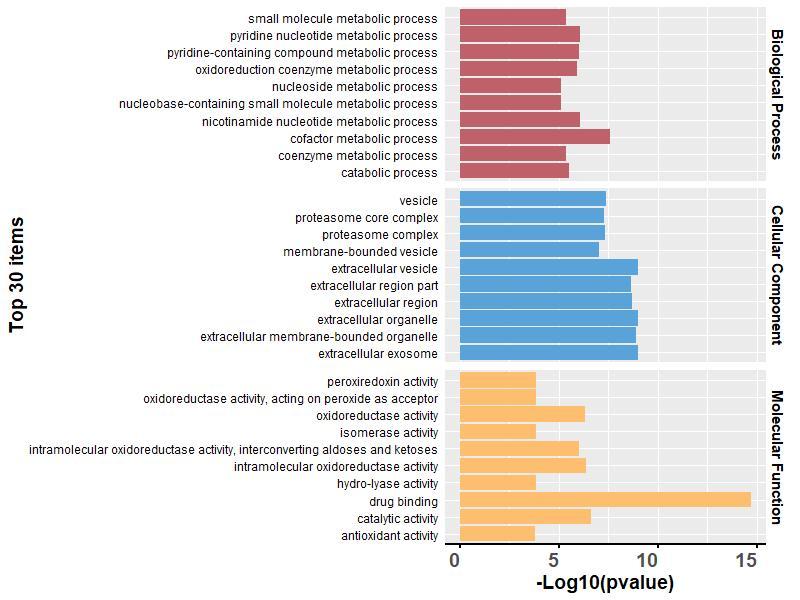


Figure 3 GO analysis of TP3vsTP4 up-regulated proteins, and GO analysis of TP3vsTP4 down-regulated proteins.


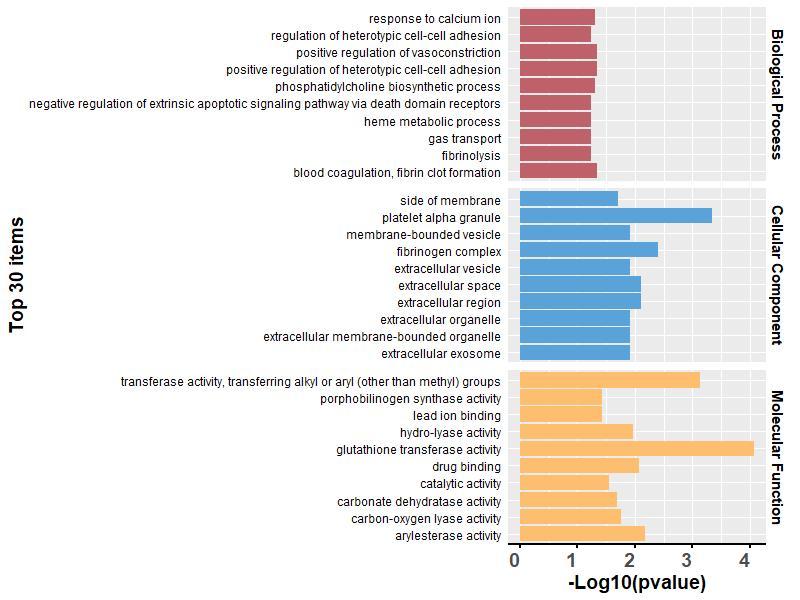

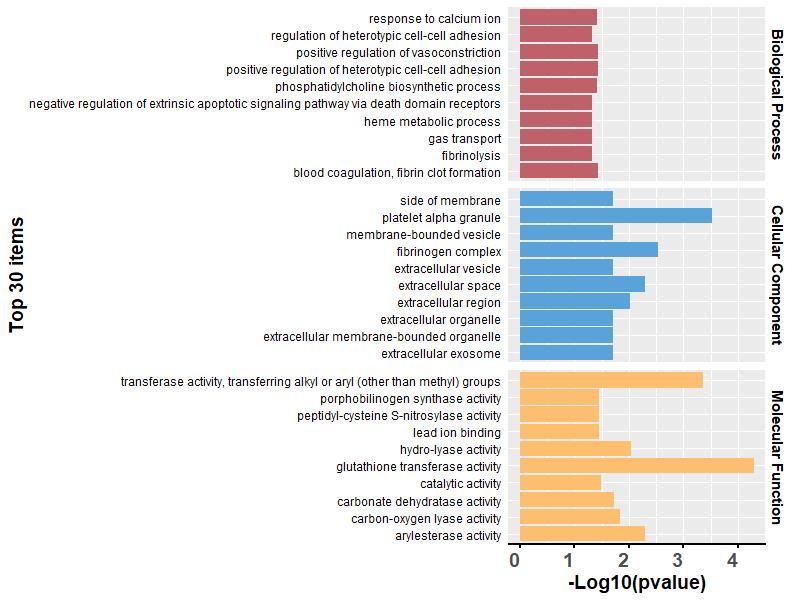


Figure 4 GO analysis of TP4vsTP5 up-regulated proteins, and GO analysis of TP4vsTP5 down-regulated proteins.


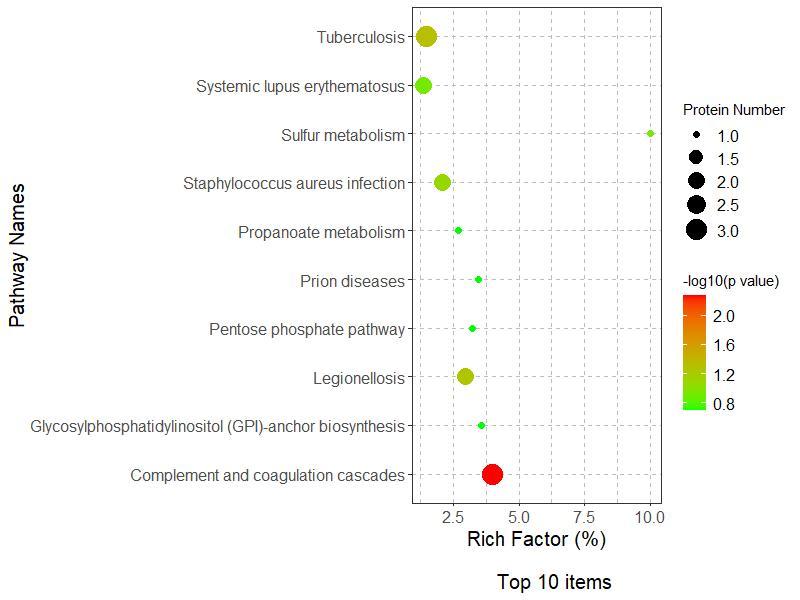

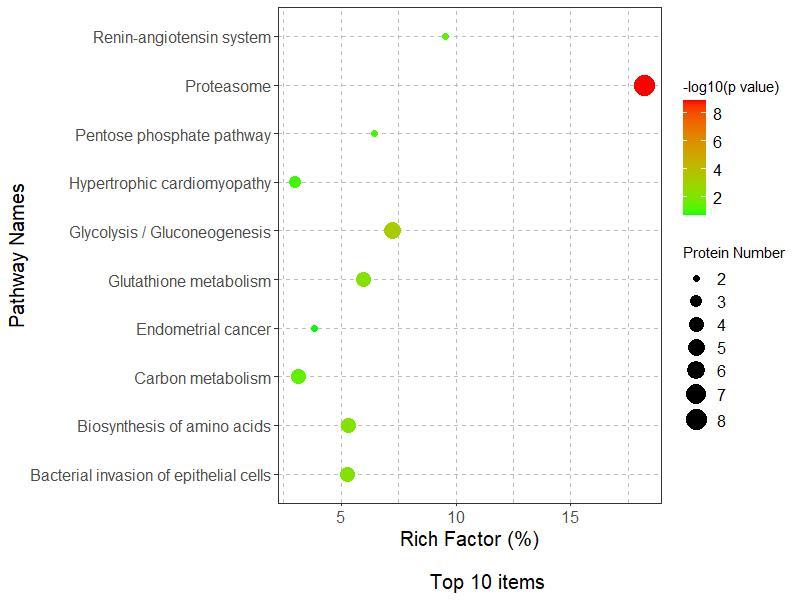


Figure 5 KEGG analysis of TP1vsTP2 up-regulated proteins, and KEGG analysis of TP1vsTP2 down-regulated proteins.


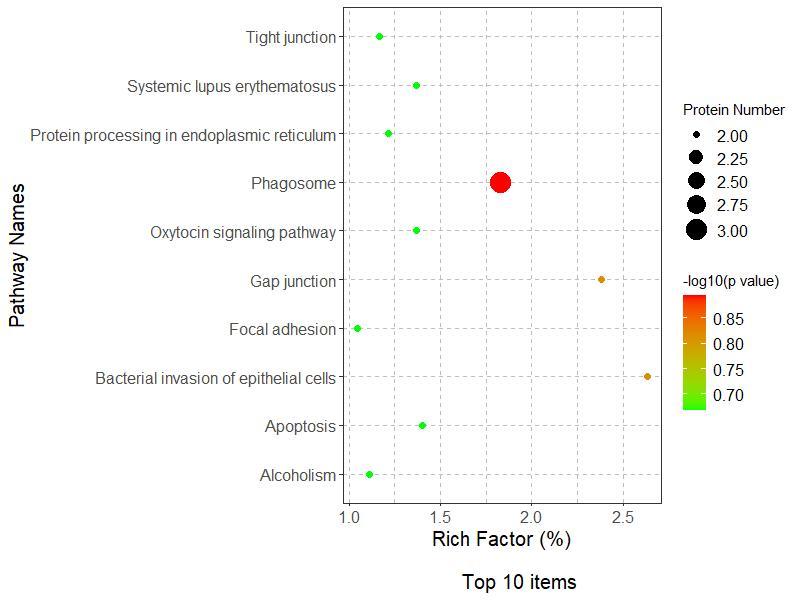


Figure 6 KEGG analysis of TP2vsTP3 up-regulated proteins


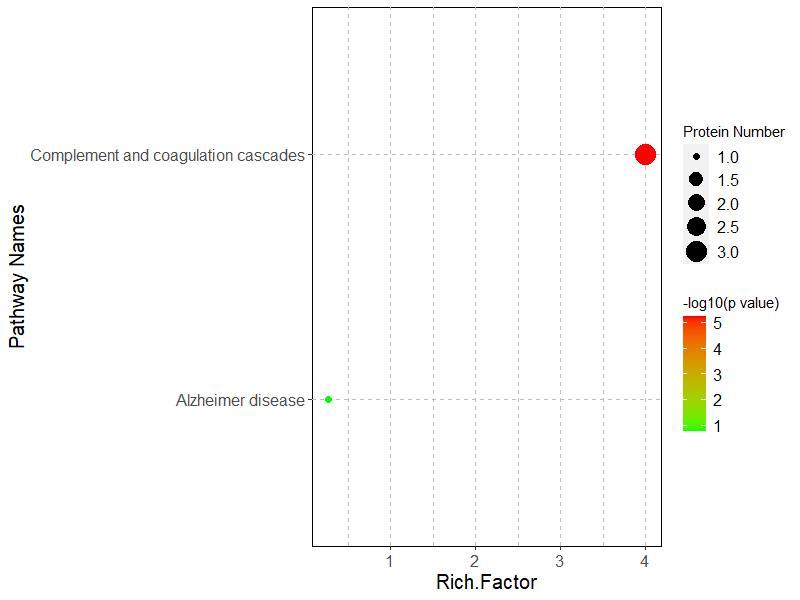

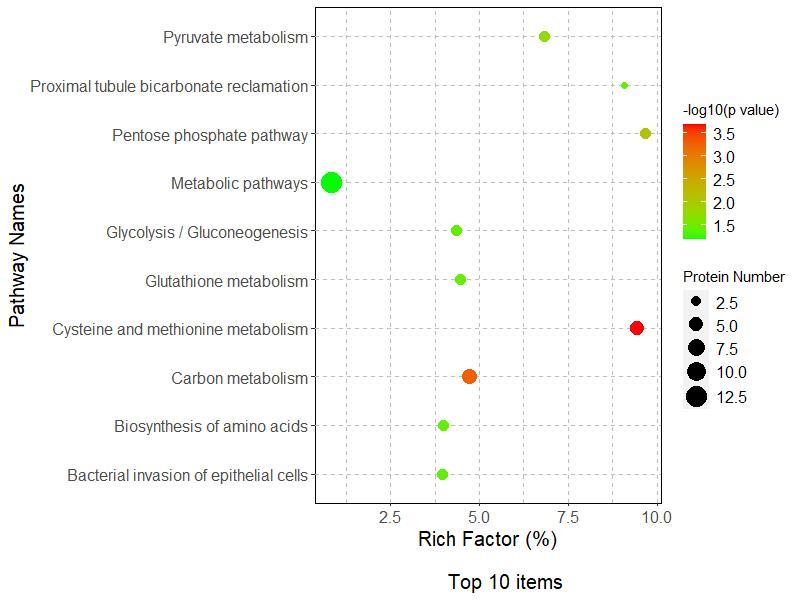


Figure 7 KEGG analysis of TP3vsTP4 up-regulated proteins, and KEGG analysis of TP3vsTP4 down-regulated proteins.


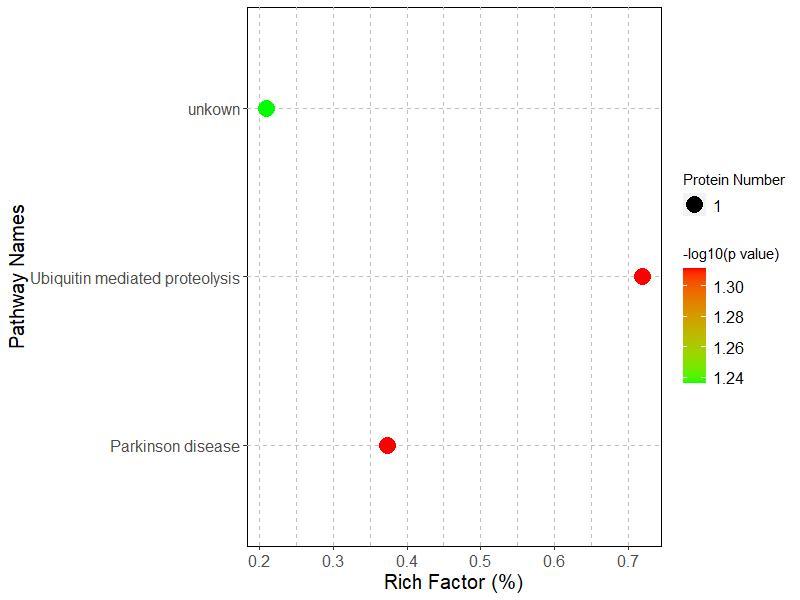

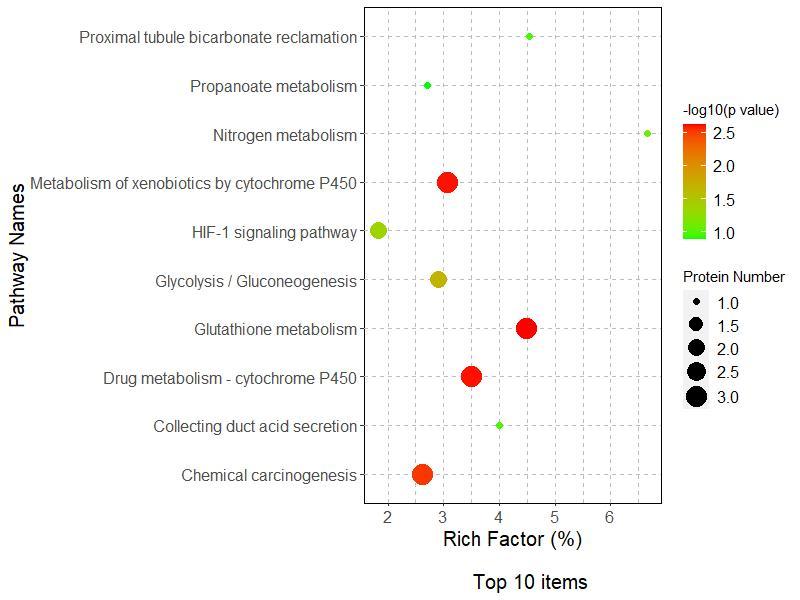


Figure 8 KEGG analysis of TP4vsTP5 up-regulated proteins, and KEGG analysis of TP4vsTP5 down-regulated proteins.


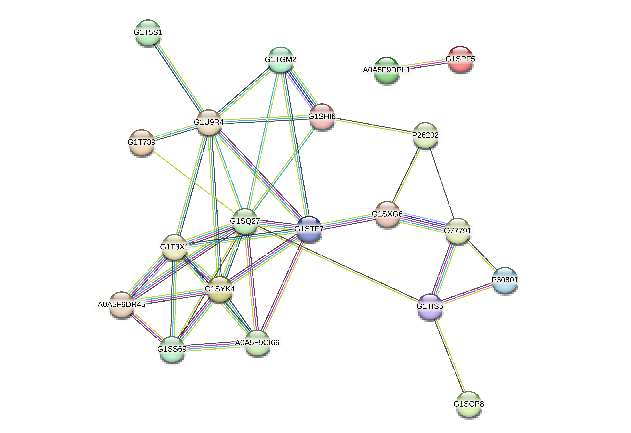

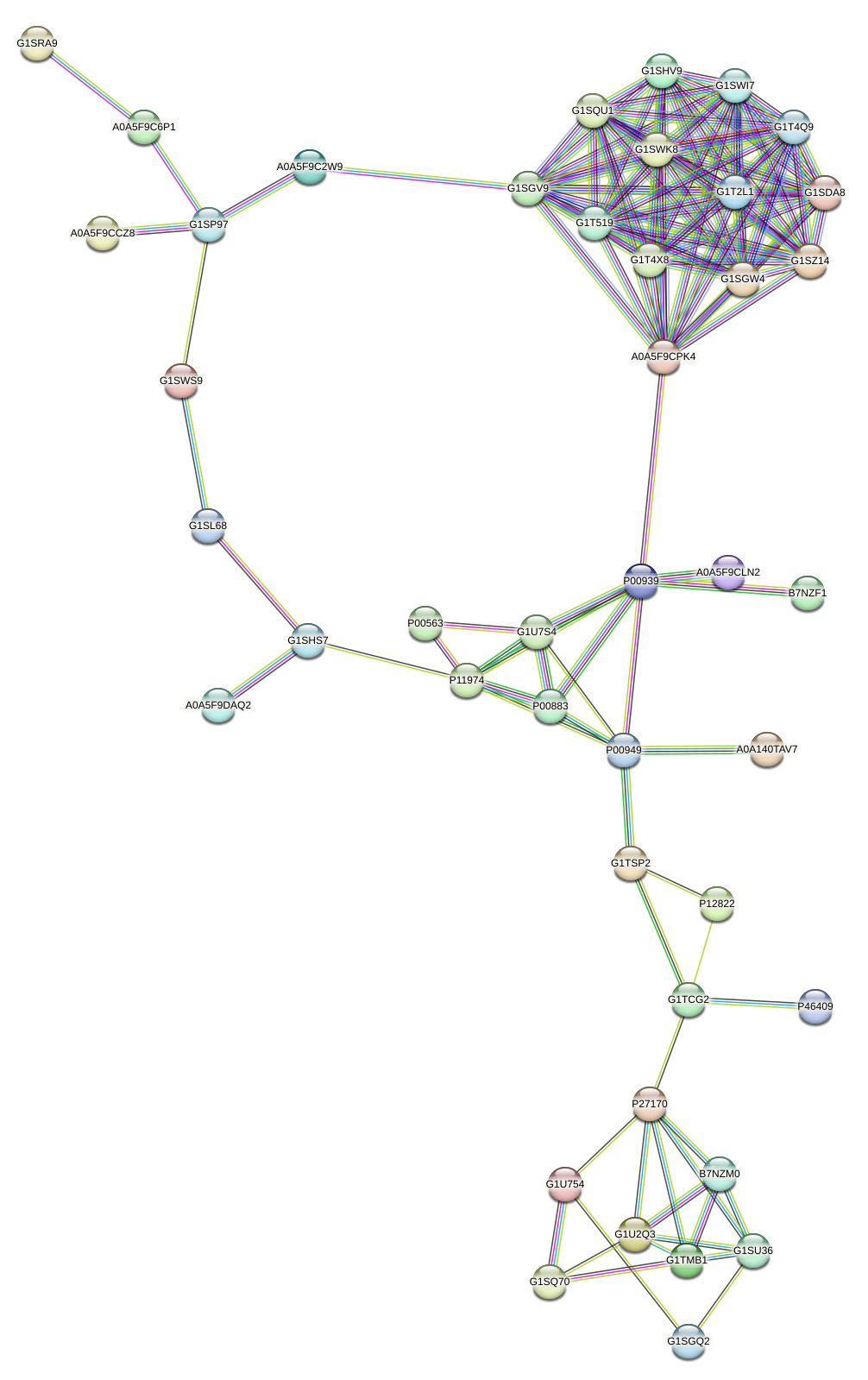


Figure 9 PPI analysis of the TP1vsTP2 up-regulated proteins, and PPI analysis of the TP1vsTP2 down-regulated proteins.


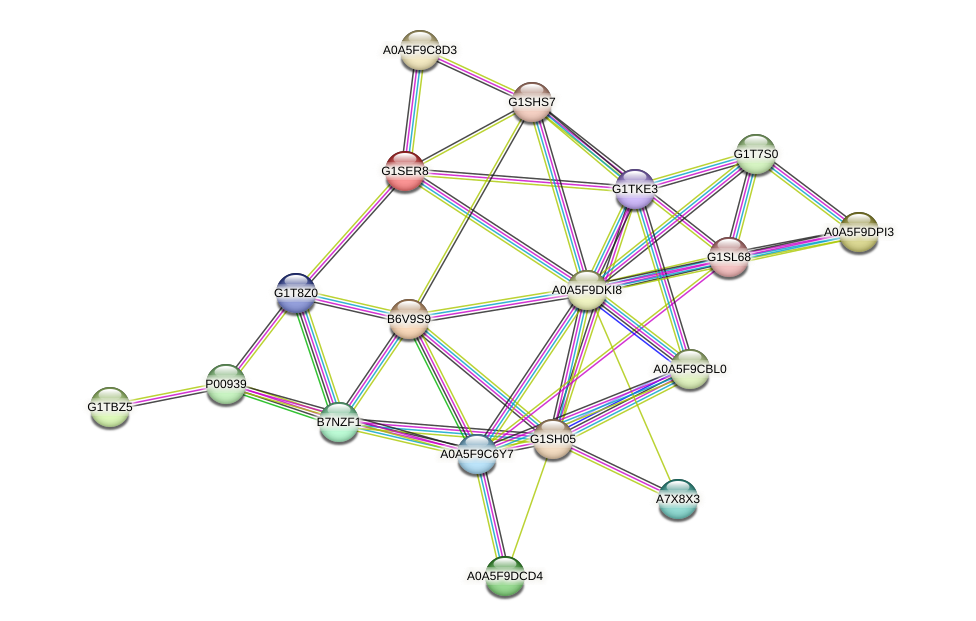


Figure 10 PPI analysis of the TP2vsTP3 up-regulated proteins.


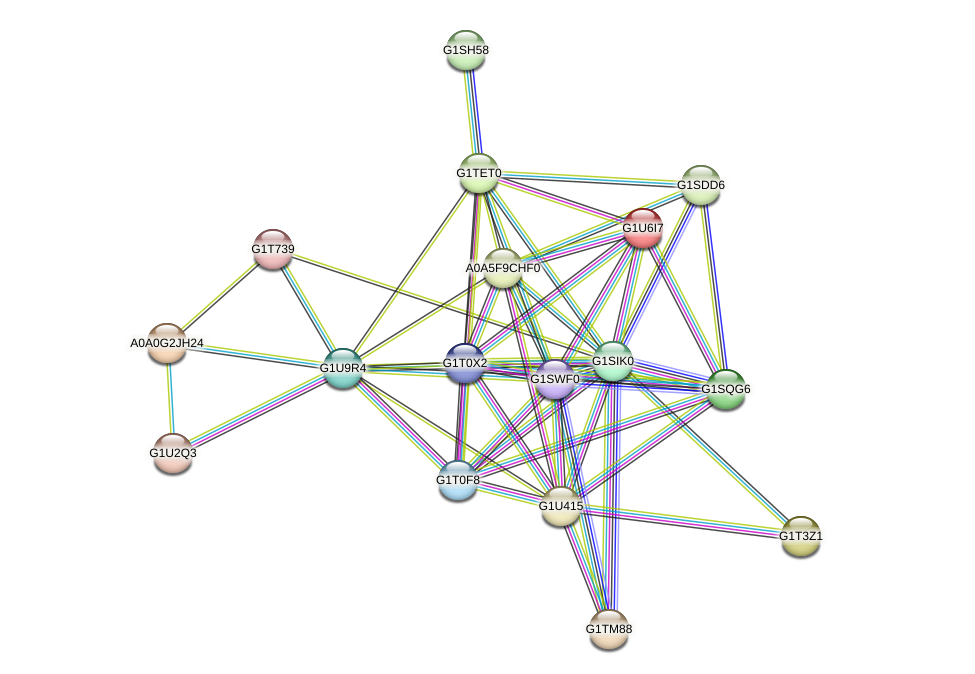

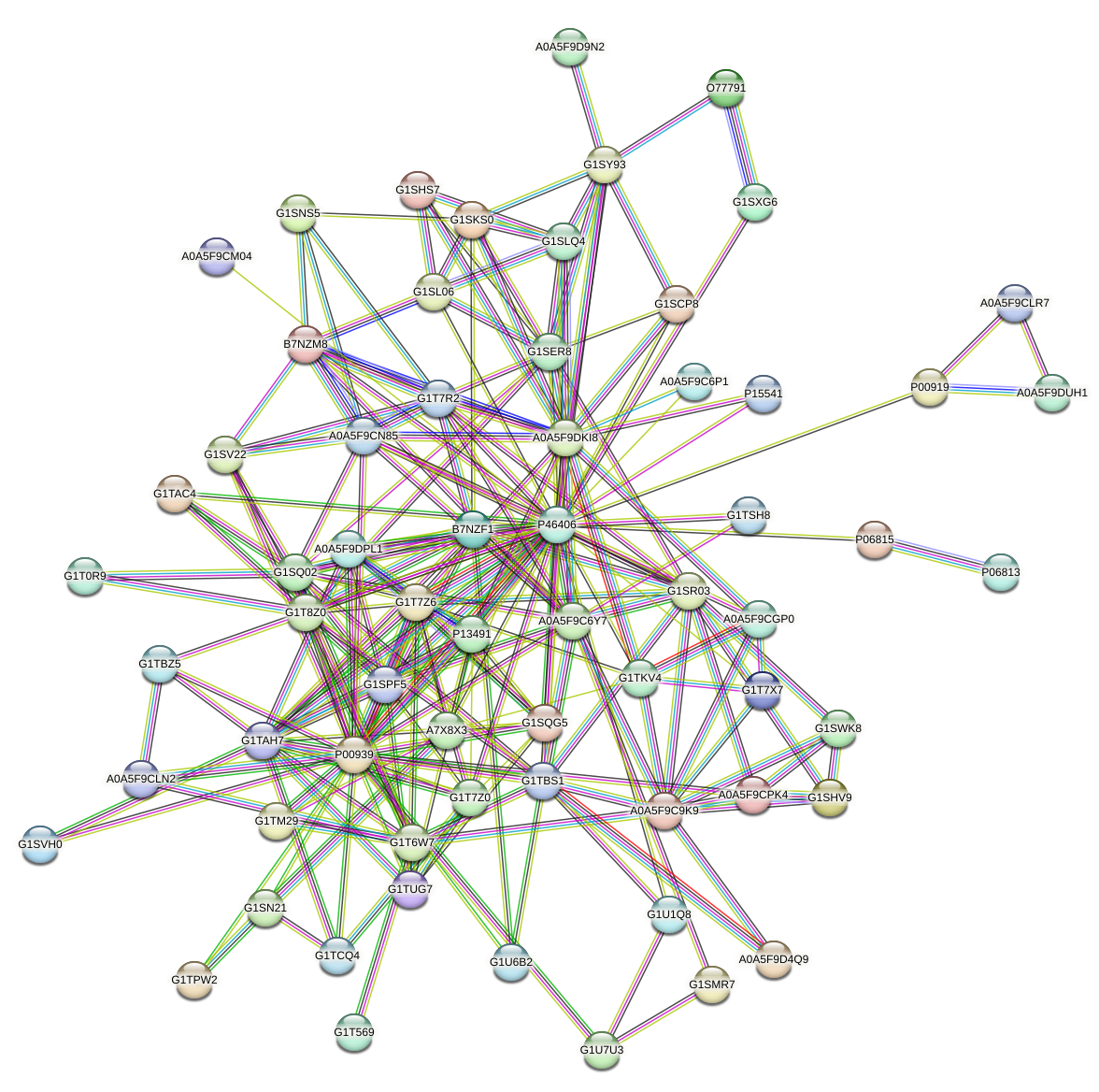


Figure 11 PPI analysis of the TP3vsTP4 up-regulated proteins, and PPI analysis of the TP3vsTP4 down-regulated proteins.


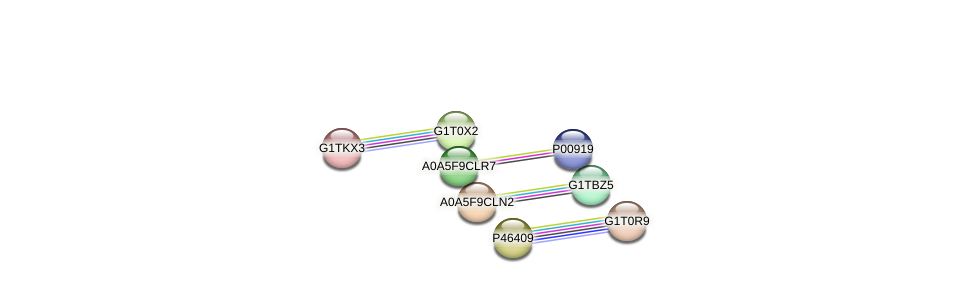


Figure 12 PPI analysis of the TP4vsTP5 down-regulated proteins.


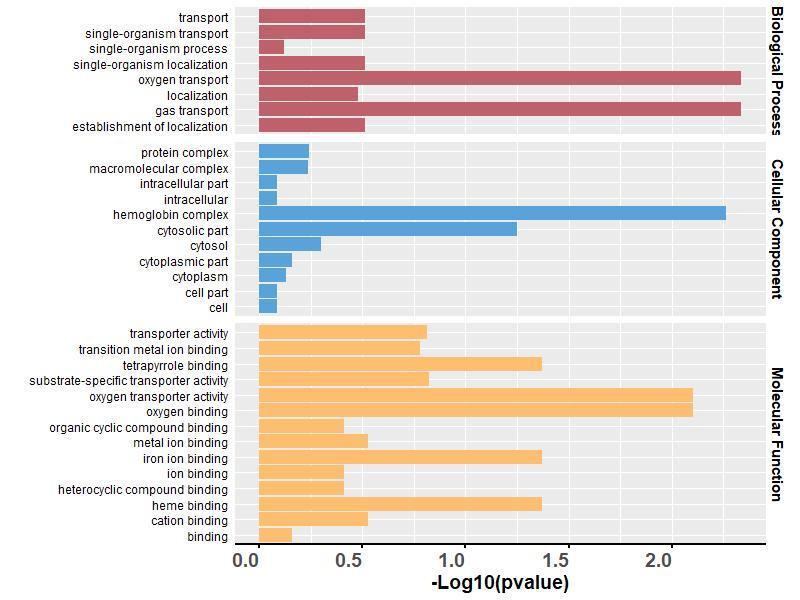


Figure 13 GO analysis of cluster1 differential proteins.


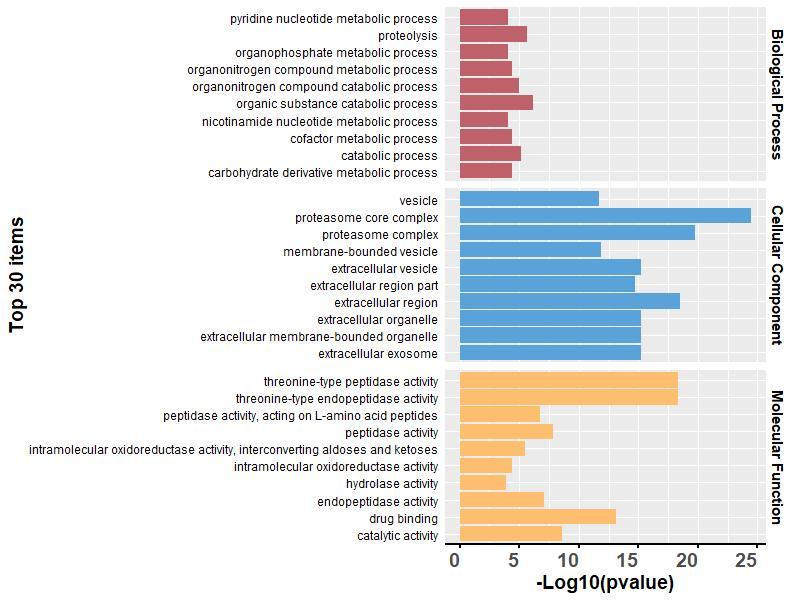


Figure 14 GO analysis of cluster2 differential proteins.


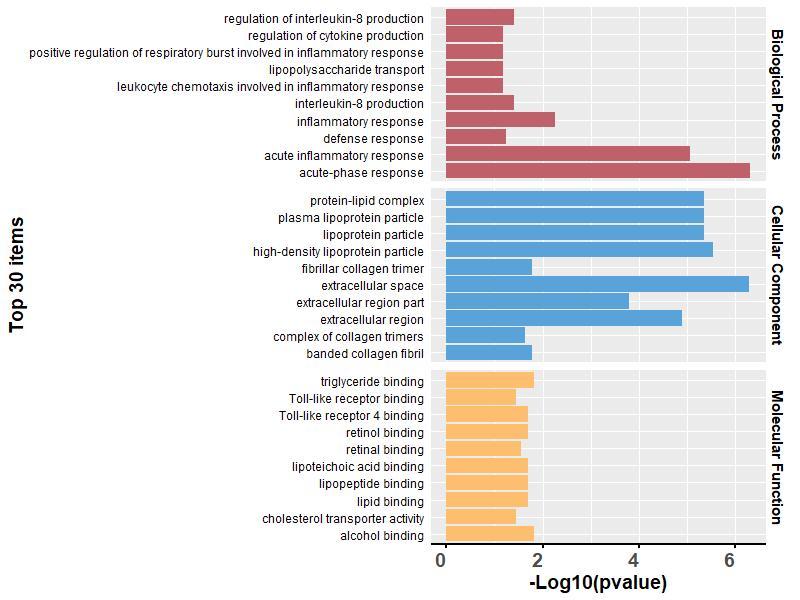


Figure 15 GO analysis of cluster3 differential proteins.


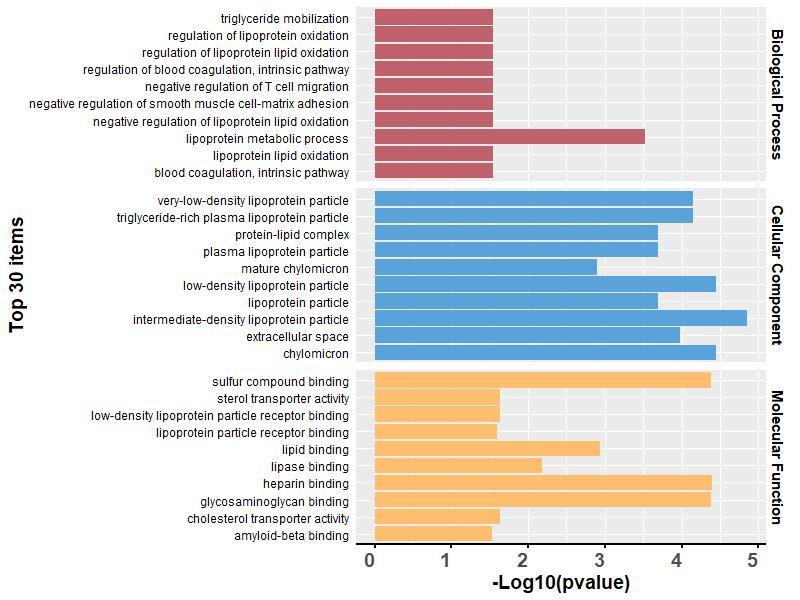


Figure 16 GO analysis of cluster4 differential proteins.


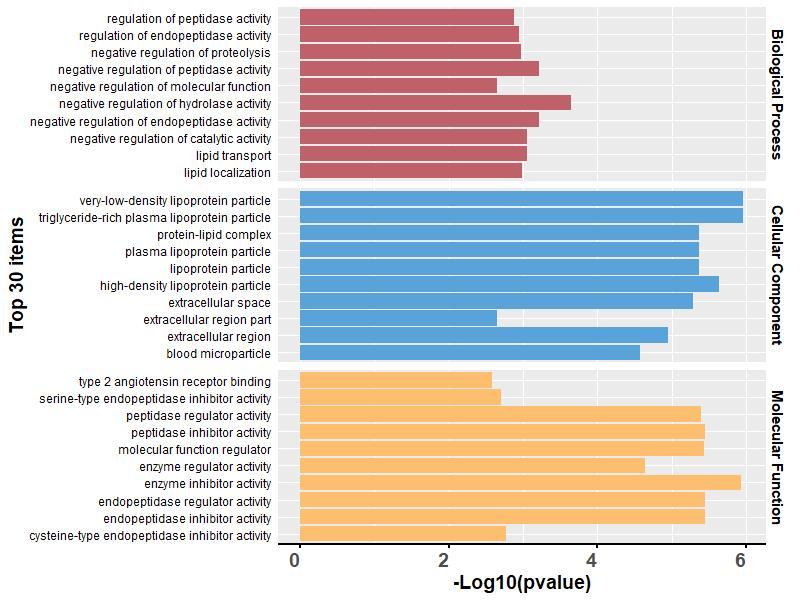


Figure 17 GO analysis of cluster5 differential proteins.


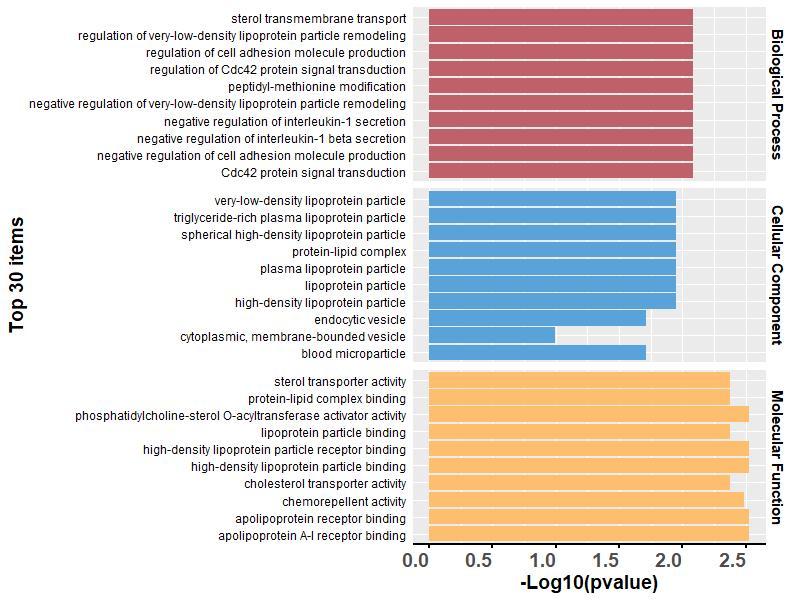


Figure 18 GO analysis of cluster6 differential proteins.


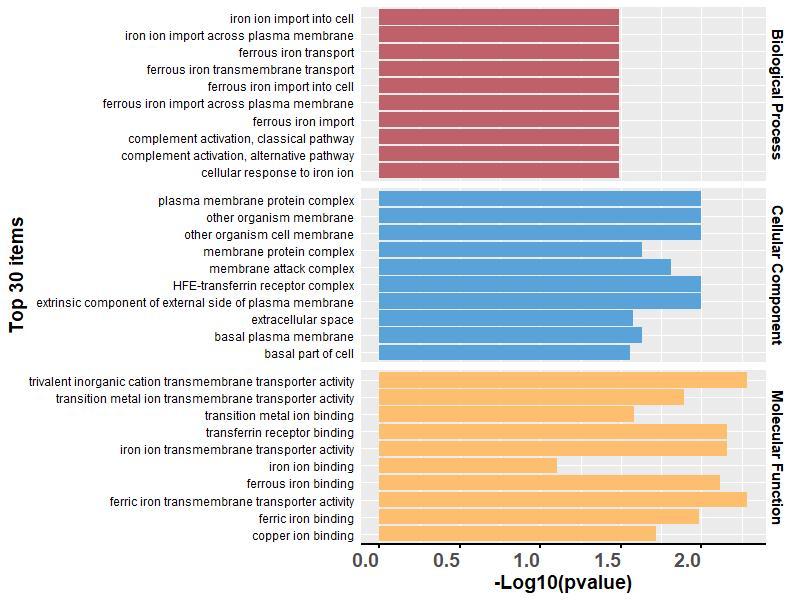


Figure 19 GO analysis of cluster7 differential proteins.


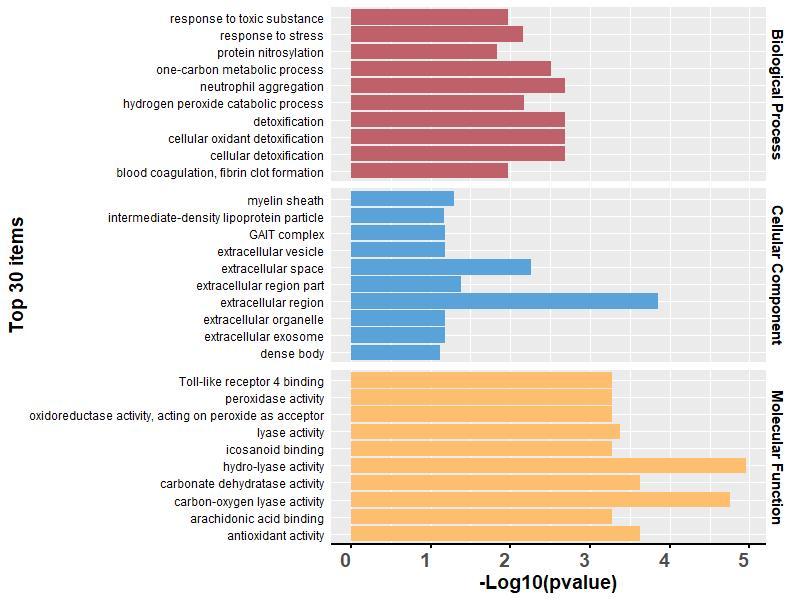


Figure 20 GO analysis of cluster8 differential proteins.
